# Optimization of Ultrasonic Dispersion of Single-Walled SWCNT Inks for Improvement of Thermoelectric Performance in SWCNT Films Using Heat Source-Free Water-Floating SWCNT Thermoelectric Generators

**DOI:** 10.3390/ma18143339

**Published:** 2025-07-16

**Authors:** Yutaro Okano, Shuya Ochiai, Hiroto Nakayama, Kiyofumi Nagai, Masayuki Takashiri

**Affiliations:** 1 Department of Materials Science, Tokai University, 4-1-1 Kitakaname, Hiratsuka 259-1292, Kanagawa, Japan; okn.yu329@gmail.com (Y.O.); 5cajm013@tokai.ac.jp (S.O.); 5cajm035@tokai.ac.jp (H.N.); 2 FUTURE I WORKS, 3-3-13 Nishishinjuku, Shinjuku 160-0023, Tokyo, Japan; knagai@future-iworks.com

**Keywords:** SWCNTs, thermoelectric generators, ultrasonic dispersion, rheometer, rheo-impedance

## Abstract

Single-walled carbon nanotube (SWCNT) inks were prepared by mixing SWCNTs with ethanol and varying the amplitude of ultrasonic dispersion. When the SWCNT inks were prepared by dispersion amplitudes at 60% (nominal value of 200 W), the SWCNT inks had low viscosity and a small variation of the particle size. The SWCNT films fabricated under this dispersion condition had well-distributed SWCNT bundles and exhibited the highest power factor. However, when the dispersion amplitude was excessive, the viscosity of the SWCNT ink increased due to the reduced contact between the SWCNTs owing to over-dispersion, and the crystallinity of the SWCNT films decreased, exhibiting a lower power factor. When the optimized SWCNT films at 60% were applied to heat-source-free water-floating SWCNT-TEGs, an output voltage of 2.0 mV could be generated under sunlight irradiation. These findings are useful for preparing various electronic devices with SWCNT films to improve the film quality using ultrasonic dispersion.

## 1. Introduction

Expectations for energy harvesting are growing annually, and remarkable progress has been made in this technology. Energy harvesting is a technology that harvests low levels of energy from the environment in various forms, such as light [1,2], vibrations [3,4], heat [5,6], and electromagnetic waves [7,8], and converts them into electricity. The power obtained by energy harvesting ranges from microwatts to milliwatts. Therefore, this technology does not provide a large amount of power; however, it can be used as a self-powered supply for small electronic devices [9]. In the Internet of Things (IoT) and cyber-physical systems (CPS), energy harvesting is a key technology for realizing autonomous power sources for wireless sensors [10,11].

Among the energy-harvesting technologies, thermoelectric generators (TEGs), which utilize thermal energy, are the most convenient because heat energy is ubiquitous and largely independent of time [12,13]. As an autonomous power source for wireless sensors, TEGs must be small, lightweight, and flexible. Flexible thin-film TEGs satisfy these requirements [14,15,16,17]. Bismuth telluride-based alloys have been investigated as thermoelectric materials for autonomous power supply applications because of their superior performance at approximately 300 K [18,19]. However, these alloys contain heavy metals that are harmful to the human body and pose significant environmental burdens. Furthermore, the reserves of alloying elements are unevenly distributed, and their quantities are limited. In particular, research is progressing on thermoelectric materials, such as organic materials and single-walled carbon nanotubes (SWCNTs), which have a low environmental impact and can be mass-produced [20,21]. In addition, organic materials and SWCNTs have the advantages of being lightweight and flexible, which are beneficial for autonomous power sources for wireless sensors [22,23,24,25,26].

In particular, SWCNTs have great potential for applications because of their unique structures [27,28,29,30]. SWCNTs consist of a single graphene sheet seamlessly wrapped in a cylindrical tube and exhibit either metallic or semiconducting properties, depending on the diameter and helicity of the tubes [31,32,33]. According to their semiconducting properties, SWCNTs exhibit p-type properties because of the adsorption of oxygen molecules on their surfaces [34,35,36,37,38]. Most SWCNT-based applications employ films consisting of large amounts of SWCNTs bound together by van der Waals forces to form SWCNT bundles. Therefore, their performance in applications, such as autonomous power sources for wireless sensors, depends on the thermoelectric properties of the SWCNT films. Generally, SWCNT films are prepared via wet processes using SWCNT inks combined with SWCNTs and solvents. Consequently, to improve the thermoelectric properties of SWCNT films, the characteristics of the SWCNT inks should be optimized. Recently, the structural, chemical, and rheological properties of nanotube particle inks have been analyzed to improve the performance of functional materials containing these particles [39,40,41]. However, the relationship between the thermoelectric properties of SWCNT films and the characteristics of SWCNT inks remains poorly understood.

In this study, SWCNT inks were prepared using ultrasonic dispersion. The relationship between the thermoelectric properties of the SWCNT films and the characteristics of the SWCNT inks was investigated. In the case of SWCNTs, changing the ultrasonic dispersion conditions affected the state of the SWCNT bundles [42,43,44,45]. Therefore, by optimizing the ultrasonic dispersion conditions of SWCNT inks, the thermoelectric properties of SWCNT films can be improved by changing the film structure. The characteristics of the SWCNT inks were investigated by a laser diffraction particle size analyzer and a rotational rheometer with an LCR meter [46]. Subsequently, the SWCNT films were fabricated using SWCNT inks via vacuum filtration. The microstructure and thermoelectric properties of the SWCNT films were investigated using various analytical methods. Finally, to apply the developed SWCNT films, heat-source-free water-floating SWCNT-TEGs [47], which generate electricity via the Seebeck effect for a self-generating temperature gradient by evaporative cooling, were fabricated, and their performance was evaluated.

## 2. Materials and Methods

**Figure 1:**
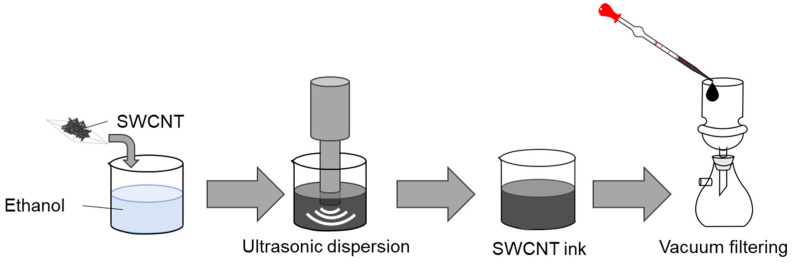
Fabrication processes of SWCNT ink via ultrasonic dispersion system and SWCNT films via vacuum filtering.

Figure 1 illustrates the manufacturing process of SWCNT inks and SWCNT films. The SWCNTs (ZEONANO SG101, ZEON, Tokyo, Japan) used in this study had diameters in the range of 3–5 nm and lengths on the order of micrometers. The SWCNT inks were prepared by mixing 80 mg of SWCNT powder with 40 mL of ethanol (Fujifilm Wako Pure Chemical, Osaka, Japan). To prepare the SWCNT inks, a probe sonicator (Branson, Ultrasonic Sonifier 250, Danbury, CT, USA) was used at a frequency of 20 kHz, ultrasonic amplitude of 21–145 μm, and ultrasonic horn tip diameter of 12.7 mm. The amplitude was calibrated based on the manufacturer’s manual. To control dispersion conditions of the SWCNT inks, the ultrasonic dispersion amplitude was varied from 10% to 90% (nominal value of 200 W) for 30 min in an ice bath. We found that the ethanol and SWCNTs separated, and the colloidal stability of the inks was insufficient when the dispersion amplitude was less than 30%. As the dispersion amplitude increased, the inks showed high colloidal stability. There was no aggregation or precipitation of the SWCNTs, even after being left for a long time.

The particle size distribution in SWCNTs after ultrasonic dispersion was determined using a laser diffraction particle size analyzer (Anton Paar, PSA 1190 L/D, Graz, Austria). The rheology of SWCNT inks was examined by a rotational rheometer (Anton Paar, MCR102e, Graz, Austria) using cone-plate geometry (Ø 50 mm) at a gap size of 0.5 mm, where the range of shear rate was 0.1–1000 s^−1^. The measurements were conducted at a constant temperature (20 °C) controlled by the Peltier system. Rheo-impedance measurements of the SWCNT inks were performed using a rotational rheometer (Anton Paar, MCR102e, Graz, Austria) with the same setting conditions as the rheology measurement and LCR meter (HIOKI, IM3536, Nagano, Japan) with a frequency range of 4–8 MHz and an applied voltage of 0.5 V.

SWCNT films were fabricated using these inks via vacuum filtration. In this process, 10 mL of SWCNT ink was drawn with a pipette and distributed uniformly onto a membrane filter (PTFE, 90 mm in diameter, ADVANTEC, Tokyo, Japan) positioned on a filter holder in a suction bottle. Filtration was accomplished by reducing the pressure within the suction bottle for 1 h using a rotary pump. This step was repeated four times to create SWCNT films with 80 mm diameter and thicknesses ranging from 50 to 100 µm, depending on the dispersion conditions of the inks. After air-drying for 24 h, the films were detached from the membrane filters.

The microstructures of the SWCNT films were analyzed by field-emission scanning electron microscope (FE-SEM; Hitachi, S-4800, Toyko, Japan). The crystallinity of the films was evaluated using a Raman microscope with an Ar+ laser beam at an excitation wavelength of 514.5 nm (XploRA, HORIBA, Kyoto, Japan). The in-plane thermoelectric properties of the SWCNT films were evaluated at ~300 K. The in-plane Seebeck coefficient, *S*, was determined at approximately 300 K using a custom-built apparatus with an accuracy of ±5% [48]. One end of the film was affixed to a heat sink, whereas the other end was attached to a Peltier module (Z-MAX, FPH1-12704AC, Tokyo, Japan). Two K-type thermocouples (diameter: 0.1 mm) were affixed to the center of the SWCNT films at a distance of 13 mm. The temperature difference between the thermocouples was varied from 0 to 4 K by controlling the electric current of the Peltier module using a DC power supply (Kikusui, PAB32-2, Yokohama, Japan), and the thermoelectric voltage was recorded at intervals of 1 K (temperature reader: KEYENCE; GR-3500, Osaka, Japan and digital multimeter: ADVANTEST, R6561, Tokyo, Japan). The Seebeck coefficient was determined from the slope of the thermoelectric voltage to the temperature difference using a linear approximation. The Seebeck coefficient was measured eight times for each sample, and the obtained values were averaged. The in-plane electrical conductivity, *σ*, was determined at approximately 300 K through the implementation of a four-point probe method (Napson, RT-70V, Tokyo, Japan), with an accuracy of ±3%. Electrical conductivity was measured eight times for each sample, and the obtained values were averaged. The in-plane power factor, *PF*, which is a prime parameter for assessing thermoelectric performance, was calculated using the measured Seebeck coefficient, *S*, and electrical conductivity, *σ*, as follows: *PF* = *σS*^2^.

## 3. Results and Discussion

### 3.1. Characteristics of SWCNT Inks

**Figure 2:**
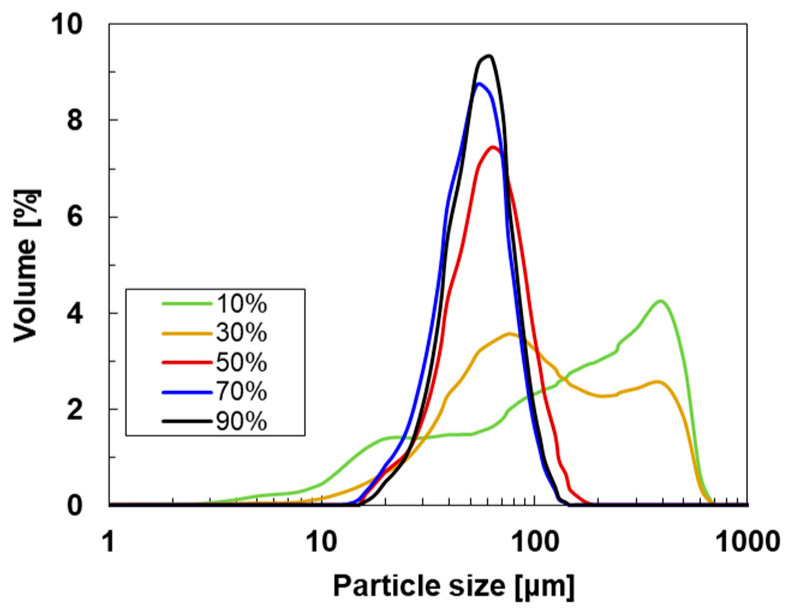
Size distribution of the SWCNT inks with different ultrasonic dispersion amplitudes.

To investigate the size of the SWCNTs, the particle size distributions of the SWCNTs were determined via integrated light scattering using a laser diffractometer based on the Fraunhofer model. The Fraunhofer model assumes spherical particle shapes and does not consider phenomena such as the absorption, refraction, reflection, or scattering of light [49]. For SWCNTs, the equivalent circular shape is measured, which includes the bundle diameter [50]. Each measurement was repeated three times for each SWCNT ink under identical conditions to obtain an average value. Figure 2 shows the size distribution of the SWCNT inks with different ultrasonic dispersion amplitudes. When the SWCNT inks were prepared at the dispersion amplitudes of 10% and 30%, the variations of the particle size (interpreted as the hydrodynamic volume) were significantly larger in the range from 5 to 700 µm. This indicates that the SWCNT inks at 10% and 30% were not sufficiently dispersed. At a dispersion amplitude of 50%, the variation of the particle size became smaller, and the median value was exhibited at approximately 70 µm. As the dispersion amplitude was further increased, the variation in particle size was further reduced, and the median value decreased to approximately 60 µm, while these characteristics did not change at dispersion amplitudes between 70% and 90%. Although the aggregates were separated into smaller fractions by the higher amplitude of ultrasonic dispersion, they were still small bundles of SWCNTs. Note that the size distribution of the SWCNT ink at a dispersion amplitude of 60% was not measured in this study, even though the highest thermoelectric performance was observed in the SWCNT film using the SWCNT ink at a dispersion amplitude of 60%, as described later in Section 3.2. Here, we calculated the average size distribution of the SWCNT inks at dispersion amplitudes of 50% and 70%. This was considered the size distribution of the SWCNT ink at a dispersion amplitude of 60%. Therefore, the SWCNT ink at a dispersion amplitude of 60% was expected to have a median particle size of approximately 60 µm, and the variation in particle size should be similar to that of the SWCNT inks at dispersion amplitudes of 50% and 70%.

**Figure 3:**
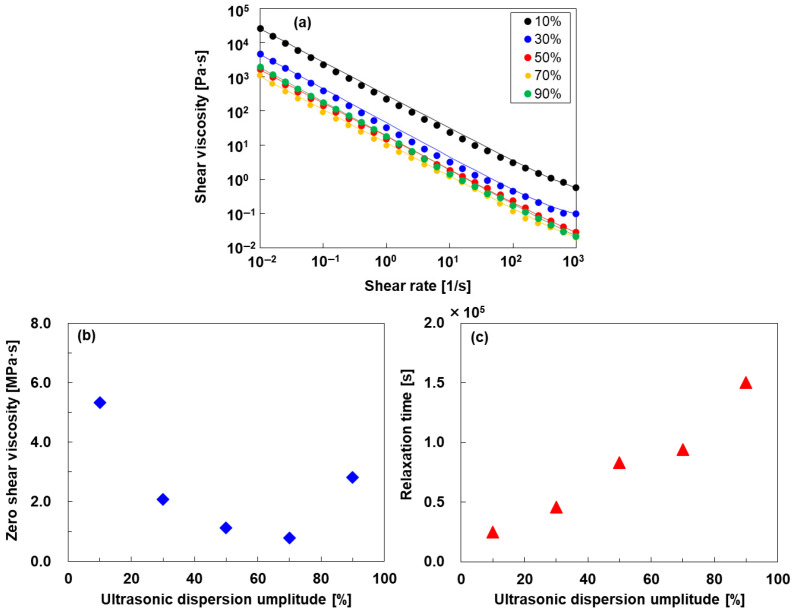
(**a**) Relationship between viscosity and shear rate using SWCNT inks with different ultrasonic dispersion amplitudes. (**b**) Zero shear rate viscosity and (**c**) relaxation time of SWCNT inks as a function of ultrasonic dispersion amplitude. The lines in (**a**) represent the fitting according to the Cross-model Equation (1).

The rheological properties of the SWCNT inks were measured using a rotational rheometer in a controlled shear rate test mode. Figure 3a shows viscosity as a function of the shear rate for the SWCNT inks in the shear rate range from 0.01 to 1000 s^−1^ on a log-log scale. All SWCNT inks displayed a typical shear-thinning behavior that is characteristic of a decrease in viscosity with increasing shear rate because the SWCNT structures in the inks start to break at high shear rates, and all the inks behave like non-Newtonian fluids.

A more quantitative analysis of the viscosity *η* vs. shear rate *γ* trends was performed using the Cross fit model, which was expressed as the following equation [51]:

(1) $$\eta =\eta_{\infty }+\frac{\left(\eta_{0}-\eta_{\infty }\right)}{1+{\left(\alpha \gamma \right)}^{m}}$$

where *η*_0_ and *η*_∞_ are the asymptotic viscosity values for low and high shear rate, and *α* represents the relaxation time, and *m* is a dimensionless parameter related to the degree of dependence of *η* on *γ* in the shear-thinning region. Specifically, *m* equals 0 and 1 can be attributed to Newtonian and plastic fluids, respectively, while *m* ranging between 0 and 1 reflects non-Newtonian pseudoplastic characteristics. In Figure 3b, we plotted the zero shear viscosity as a function of the various ultrasonic dispersion amplitudes. The SWCNT inks with nonuniform dispersions at amplitudes of 10% exhibited high viscosities. The zero shear viscosity of the SWCNT inks decreased until the dispersion amplitude reached 70% because the SWCNT bundles loosened and the dispersion progressed, which is consistent with the results of the particle size measurements as shown in Figure 2. Further increasing the dispersion amplitude to 90%, the viscosity of the SWCNT inks increased because the specific surface area was increased owing to over-dispersion [52]. Figure 3c shows the relaxation time of SWCNT inks prepared using different ultrasonic dispersion amplitudes. The relaxation time linearly increased with increasing dispersion amplitude. The reciprocal of relaxation time (*α*) represents the critical shear rate (γ*), which is related to the transition from the Newtonian to the non-Newtonian regime. Therefore, the lowest critical shear rate of 6.5 × 10^−6^ s^−1^ was exhibited at a dispersion amplitude of 90%, while the lowest critical shear rate of 4.0 × 10^−5^ s^−1^ was exhibited at a dispersion amplitude of 10%.

**Figure 4:**
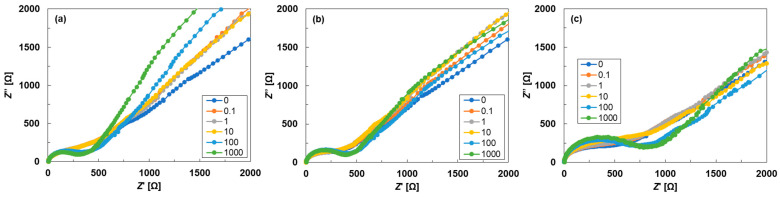
Nyquist plots of rheo-impedance measured using SWCNT inks prepared with ultrasonic dispersion amplitudes of (**a**) 50%, (**b**) 70%, and (**c**) 90%, while varying the shear rates of 0, 0.1, 1, 10, 100, and 1000 s^−1^.

Figure 4 shows the Nyquist plots of the rheo-impedance measured using SWCNT inks prepared with different ultrasonic dispersion amplitudes. Note that SWCNT inks with dispersion amplitudes of 10% and 30% were not measured due to insufficient dispersion, as shown in Figure 2. In Figure 4a, the SWCNT inks with a dispersion amplitude of 50% showed that the straight line with a 45° slope and the semicircles were distinguishable at shear rates of 100 and 1000 s^−1^. When the shear rate was less than 10 s^−1^, the semicircle and line began to overlap. This overlap indicated that the diffusion of the active species was slower than the time constant of the charge-transfer reaction [47]. The SWCNT inks with dispersion amplitudes of 70% and 90% exhibited similar trends to those of the SWCNT ink with a dispersion amplitude of 50%. However, the radii of the semicircles in the impedance spectra increased with increasing dispersion amplitude, indicating that the charge transfer resistance increased [53]. The mechanism of the increase in the charge transfer resistance is that the over-dispersion creates gaps between SWCNTs. Hydroxyl and carboxyl groups derived from ethanol then penetrate these gaps and attach to the SWCNT surface. Consequently, these layers act as a resistive barrier that inhibits charge transfer between SWCNTs [54,55].

### 3.2. Structural and Thermoelectric Properties of SWCNT Films

**Figure 5:**
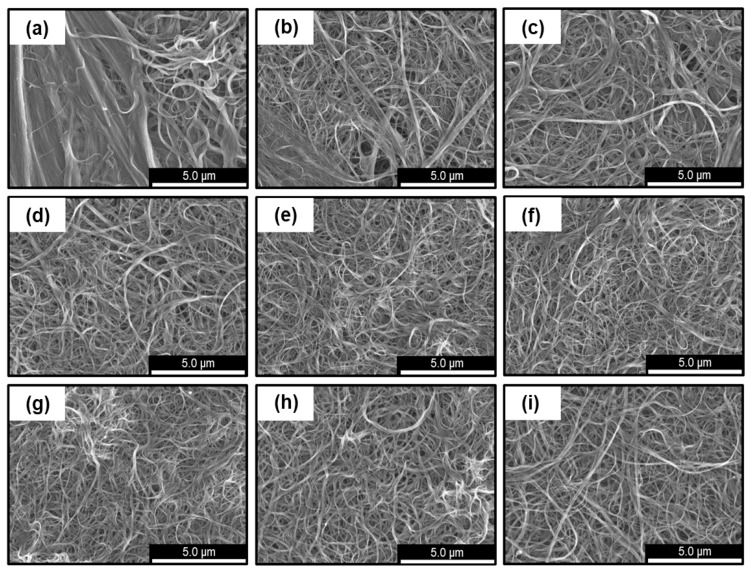
Surface morphologies of SWCNT films with different ultrasonic dispersion amplitudes in the SWCNT ink production. (**a**) 10%, (**b**) 20%, (**c**) 30%, (**d**) 40%, (**e**) 50%, (**f**) 60%, (**g**) 70%, (**h**) 80%, and (**i**) 90%.

The microstructures of the SWCNT films observed using FE-SEM are shown in Figure 5. The surface morphology of the SWCNT films varied significantly depending on the dispersion amplitude used to prepare the SWCNT inks. At the lowest power of dispersion amplitude of 10%, several thick SWCNT bundles with random orientations were observed, up to approximately 0.5 µm in diameter (Figure 5a). When the dispersion amplitude was increased to 40%, the overall bundle thickness became thinner; however, there was a large variation in bundle thickness (Figure 5b–d). When the dispersion amplitude was further increased by over 50%, the thick bundles could not be observed, and only bundles with diameters of tens of nanometers were observed (Figure 5e–i). Therefore, the surface morphology of the SWCNT films was significantly related to the characteristics of the SWCNT inks.

**Figure 6:**
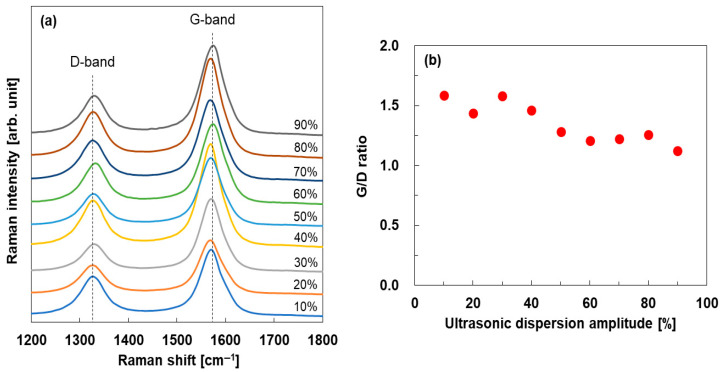
(**a**) Raman spectra of SWCNT films with different ultrasonic dispersion amplitudes in the SWCNT ink production and (**b**) *I_G_*/*I_D_* ratio of the SWCNT films.

Raman spectra of the SWCNT films produced using inks with varying ultrasonic dispersion amplitudes are shown in Figure 6a. The Raman spectra of all SWCNT films showed G- and D-bands at approximately 1590 and 1350 cm^−1^, respectively. The G-band is a graphite-derived spectrum of carbon atoms in the hexagonal lattice, whereas the D-band appears when the disorder of the carbon basal plane lattices (e.g., impurities, edges, and defects) is included in the crystal lattice of the SWCNTs. Thus, the ratio (*I_G_*/*I_D_*) between the integrated intensities of the G- and D-bands reflects the degree of structural disorder in the SWCNTs [56]. To better observe the details of Figure 6a, we plotted the *I_G_*/*I_D_* ratio as a function of the ultrasonic dispersion amplitude in Figure 6b. The *I_G_*/*I_D_* ratio of the SWCNT film with the lowest power of ultrasonic dispersion in ink production (10%) was 1.58. As the dispersion amplitude increased, the *I_G_*/*I_D_* ratio decreased linearly. At the highest dispersion amplitude of 90%, the *I_G_*/*I_D_* ratio was 1.12. Therefore, the structural disorder in the SWCNT films increases with increasing dispersion amplitude. A similar phenomenon was observed in graphene sheets subjected to the tip sonication of a graphene solvent dispersion [57].

To investigate the mechanical properties of the SWCNT films, the tensile strength of the SWCNT films was measured by tensile test. The relationship between the tensile strength of the SWCNT films and the dispersion amplitude for preparing the SWCNT inks is shown in the Supplementary Materials (Figure S1). In brief, the SWCNT films with dispersion amplitudes of 50% and 70% exhibited the highest tensile strength of approximately 18 MPa. An increase or decrease in the dispersion amplitude also reduced the tensile strength of the SWCNT films.

**Figure 7:**
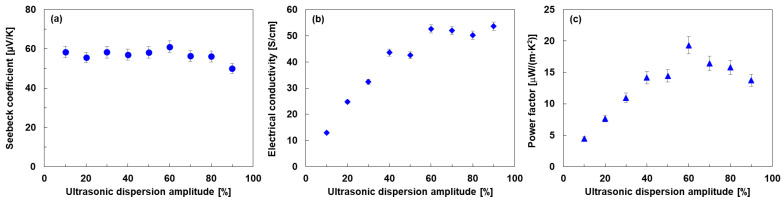
In-plane thermoelectric properties of SWCNT films with different ultrasonic dispersion amplitudes in the SWCNT ink production. (**a**) Seebeck coefficient, (**b**) electrical conductivity, and (**c**) power factor.

Figure 7 shows the thermoelectric properties of SWCNT films with different ultrasonic dispersion amplitudes for the preparation of SWCNT inks. In Figure 7a, the Seebeck coefficient of the SWCNT film remains almost constant at approximately 60 µV/K when the ultrasonic dispersion amplitude is from 10% to 60%. As the dispersion amplitude increases, the Seebeck coefficient gradually decreases. At a dispersion amplitude of 90%, the SWCNT film exhibited a Seebeck coefficient of 50 µV/K. This occurred because the carrier concentration increased at higher dispersion amplitudes. For metals or degenerated semiconductors based on parabolic band and energy-independent scattering approximation [58], the relationship between *S* and carrier concentration *n* is expressed by Equation (2) [59].

(2) $$S=\frac{8\pi^{2}{k_{B}}^{2}}{3eh^{2}}m^{*}T{\left(\frac{\pi }{3n}\right)}^{\frac{2}{3}},$$

where *k_B_*, *h*, m*, and *T* are the Boltzmann constant, Planck’s constant, effective mass, and absolute temperature, respectively. Therefore, the Seebeck coefficient is negatively correlated with the carrier concentration. When the dispersion amplitude is increased from 10% to 60% and the Seebeck coefficient is maintained at approximately the same value, the carrier concentration remains unchanged, assuming that the effective mass remains unchanged. However, the Seebeck coefficient gradually decreases with a further increase in the dispersion amplitude. A possible explanation is that the carrier concentration increased by increasing the defect density of the SWCNT films by applying a higher dispersion amplitude during SWCNT ink production [60,61], which corresponds to the results in Figure 6b. In Figure 7b, the lowest electrical conductivity of 13 S/cm was observed at an ultrasonic dispersion amplitude of 10%. The electrical conductivity of the SWCNT film increased as the dispersion amplitude was increased from 10% to 60%. When the dispersion amplitude was increased further, the electrical conductivity remained almost constant at approximately 52 S/cm. The electrical conductivity, *σ*, is expressed as Equation (3), written as follows:

(3) σ=enμ

where *μ* is mobility. As the dispersion amplitude increased from 10% to 60%, the increase in conductivity was owing to increased mobility, while the carrier concentration remained unchanged. The mechanism for the increase in mobility is the proper dispersion of the agglomerated SWCNT bundles [62], which is supported by the viscosity of the SWCNT inks shown in Figure 3 and the SEM images of the SWCNT films shown in Figure 5. In the range of 60% to 90% dispersion amplitude, the electrical conductivity becomes almost constant because the decrease in mobility and the increase in carrier concentration offset each other. The mechanism by which the mobility decreases as the dispersion amplitude increases is possibly because the SWCNTs are over-dispersed at high ultrasonic amplitudes, resulting in reduced contact between the SWCNTs. This mechanism is supported by the results of the impedance spectra shown in Figure 4, where the decrease in mobility is owing to an increase in the charge-transfer resistance. In Figure 7c, the power factor of the SWCNT films increased with increasing the dispersion amplitude from 10% to 60% because the electrical conductivity was increased, while the Seebeck coefficient remained unchanged. However, the power factor of the SWCNT films decreased with increasing the dispersion amplitude from 60% to 90% because the Seebeck coefficient was decreased, whereas the electrical conductivity remained unchanged. Consequently, the highest power factor of 19.3 μW/(m·K^2^) was exhibited at the dispersion amplitude of 60%.

### 3.3. Performance of Water-Floating SWCNT-TEGs

**Figure 8:**
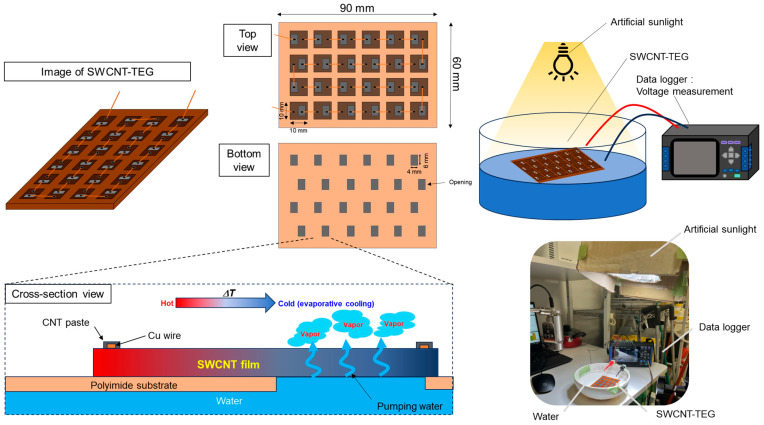
Manufacturing process and measurement procedure of water-floating SWCNT-TEG.

In this section, we fabricated the water-floating SWCNT-TEG using the SWCNT films and evaluated the performance. Figure 8 illustrates the configuration of the SWCNT-TEG and the methodology employed to assess its performance. The fabrication of the SWCNT-TEG was based on our previous report [47]. To assemble the SWCNT-TEG, the SWCNT films were cut into 24 pieces measuring 10 mm in length and 10 mm in width. The substrate, a polyimide sheet (Kapton, DuPont-Toray, Tokyo, Japan) measuring 90 mm × 60 mm and 125 µm thick, was prepared by drilling 24 rectangular holes, each measuring 6 mm × 4 mm, in a staggered arrangement. Twenty-four sections of the SWCNT films were bonded to the substrate with double-coated adhesive tape such that the adjacent films were each half-covered by a hole in the polyimide. Twenty-four SWCNT films were interconnected in a series of configurations using thin Cu wires. To measure the output voltage of the SWCNT-TEG, two copper wire electrodes were affixed to both ends of the film, and the opposite ends of the copper wire electrodes were linked to a data logger (GRAPHTEC GL240, Yokohama, Japan). A plastic container of diameter 160 mm was filled with 500 mL of water at an approximate temperature of 300 K. The SWCNT-TEG floated on the surface of the water. The performance of the SWCNT-TEG was evaluated under irradiation with a light intensity of approximately 1000 W/m^2^ using an artificial sunlight source (SERIC, SOLAX 100 W XC-100 B, Koshigaya, Japan). The distance between the sunlight source and SWCNT-TEG was 700 mm. The output voltage of the SWCNT-TEG was measured using a data logger, and the water temperature was measured using a 0.1 mm diameter K-type thermocouple with a data logger for 60 min after light exposure.

**Figure 9:**
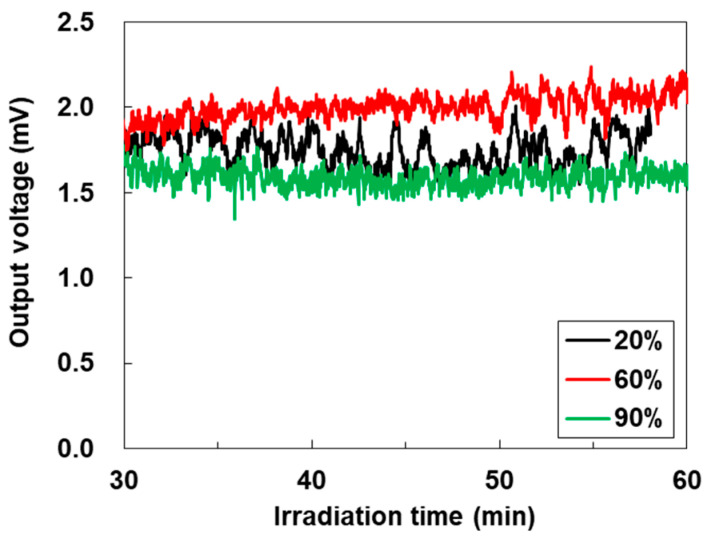
Irradiation time dependence of the output voltage in water-floating SWCNT-TEG with different ultrasonic dispersion amplitudes in the SWCNT ink production.

Figure 9 shows the time dependence of the performance of the SWCNT-TEG with different ultrasonic dispersion amplitudes in ink production after 30 min of sunlight irradiation. The output voltage of the SWCNT-TEGs remained almost stable. The highest output voltage was exhibited at the SWCNT-TEG at a dispersion amplitude of 60%, which corresponds to the power factor of the SWCNT film. The average output voltage during the irradiation period from 30 to 60 min was 2.0 mV. The temperature difference in the TEGs was calculated based on the measured output voltage and Seebeck coefficient, as shown in Figure 7a. The calculated temperature difference is approximately 1.4 K. Therefore, we obtained an output voltage from water-floating SWCNT-TEGs by creating the temperature difference. This study is useful for preparing various electronic devices with SWCNT films to improve the film quality using ultrasonic dispersion. The best performance of the water-floating SWCNT-TEG was compared to that of SWCNT-TEGs with various structures and operating conditions [48,63,64,65,66,67,68,69], as shown in Table 1. Even though the temperature difference created by the water-floating SWCNT-TEG in this study was small, its normalized output voltage was comparable to that of other SWCNT-TEGs.

## 4. Conclusions

To improve the thermoelectric properties of SWCNT films, we investigated the optimal conditions for the ultrasonic dispersion amplitude in the preparation of SWCNT inks mixed with SWCNTs and ethanol. The characteristics of the SWCNT inks were investigated using a laser diffraction particle size analyzer and a rotational rheometer with an LCR meter. The viscosity of the SWCNT inks decreased with increasing ultrasonic dispersion amplitude because of the homogeneous dispersion of the SWCNTs in ethanol. However, when the dispersion amplitude was significantly high at 90%, the viscosity of the ink increased due to the reduced contact between the SWCNTs owing to over-dispersion, which corresponded to the increase in charge transfer resistance determined from the rheo-impedance measurements. The SWCNT films were prepared using SWCNT inks via vacuum filtering. When the dispersion amplitude was increased during the SWCNT ink production, the SWCNT bundles were well distributed in the films, but the crystallinity decreased. Consequently, the power factor reached the highest value of 19.3 μW/(m·K^2^) at a dispersion amplitude of 60%. In addition, the optimized SWCNT films were applied to heat-source-free water-floating SWCNT-TEGs. The output voltage (2.0 mV) was observed under artificial sunlight irradiation.

## Figures and Tables

**Table 1:** Comparison of SWCNT-TEGs with various structures and operating conditions.

Characteristic of SWCNT-TEGs	Heat Source	Output Voltage [mV]	Output Current [mA]	Δ*T* [K]	Number of Sheets	Normalized Output Voltage [μV/(K·sheet)]	Ref.
Water-floating SWCNT-TEGs	No-use	2.0		1.4	24	60	This work
All-solid-state flexible material-based TEGs	Use	850	2.6	34			[63]
All-CNT yarns-based TEGs	Use	12		5	120	20	[64]
Slitted Kirigami Structured SWCNT TEGs	Use	9.9			6		[65]
Fully printed and flexible SWCNT TEGs	Use	1110	1.7	300	84	44	[66]
Foldable SWCNT TEGs	Use	20	0.25	10	75	27	[67]
Painted SWCNT-TEGs on Japanese paper	Use	10.4		65	6	27	[48]
Dip-coated SWCNT/mesh sheet TEGs	Use	31.5		62	8	64	[68]
Flexible SWCNT TEGs on polyimide	Use	24.0		80	8	38	[69]

## Data Availability

The original contributions presented in this study are included in the article/Supplementary Material. Further inquiries can be directed to the corresponding author.

## Supplementary Materials

The following supporting information can be downloaded at https://www.mdpi.com/article/10.3390/ma18143339/s1, Figure S1: Tensile strength of SWCNT films as a function of ultrasonic dispersion amplitude.
