# Supplementary material for: Optimization of Ultrasonic Dispersion of Single-Walled SWCNT Inks for Improvement of Thermoelectric Performance in SWCNT Films Using Heat Source-Free Water-Floating SWCNT Thermoelectric Generators
Source: Materials (Basel). 2025 Jul 16;18(14):3339. doi: 10.3390/ma18143339 (PMC12300955; doi:10.3390/ma18143339)
Supplement: Supplementary file 1 [file materials-18-03339-s001.zip › materials-3709208-supplementary.pdf]

Optimization of Ultrasonic Dispersion of Single-Walled SWCNT Inks for Improvement of Thermoelectric Performance in SWCNT Films Using Heat Source-Free Water-Floating SWCNT Thermoelectric Generators

Yutaro Okano[1], Shuya Ochiai[1], Hiroto Nakayama[1], Kiyofumi Nagai[2], and Masayuki Takashiri[1,*]

[1]Department of Materials Science, Tokai University, 4-1-1 Kitakaname, Hiratsuka 259-1292, Kanagawa, Japan

[2]FUTURE I WORKS, 3-3-13 Nishishinjuku, Shinjuku 160-0023, Tokyo, Japan

[*]Correspondence: takashiri@tokai.ac.jp

Supplementary Materials

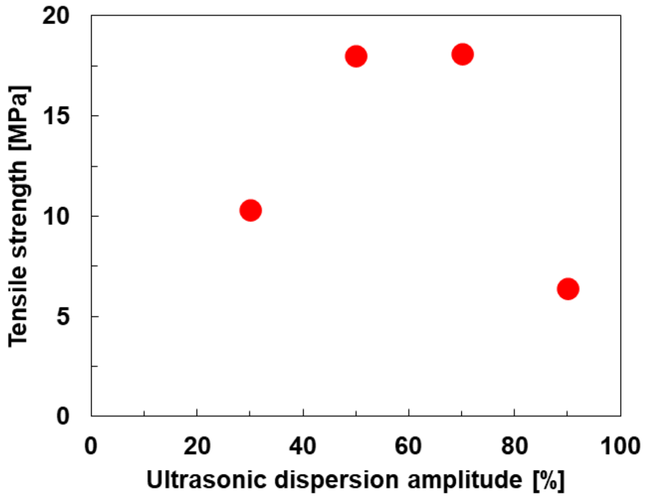

Figure S1: Tensile strength of SWCNT films as a function of ultrasonic dispersion amplitude.

To investigate the mechanical properties of the SWCNT films, the tensile strength of the SWCNT films was measured by tensile test (MX-1000N-FA, IMADA) at a temperature of approximately 300 K and a testing speed of 10 mm/min. The tensile samples were cut from the SWCNT films using a custom-made dogbone-shaped die. Figure S1 shows the tensile strength of the SWCNT films as a function of ultrasonic dispersion amplitude. The highest tensile strength of approximately 18 MPa was observed at the SWNCT films with the dispersion amplitudes of 50% and 70%. An increase or decrease in the dispersion amplitude also reduced the tensile strength of the SWCNT films.
